# Perceived ageism, macro-level sociopolitical factors, and subjective well-being: a cross-national study of older adults in 43 societies

**DOI:** 10.1093/geronb/gbaf191

**Published:** 2025-10-06

**Authors:** Xi Chen, Fei Meng

**Affiliations:** Department of Sociology and Social Policy, Lingnan University, 8 Castle Peak Road, Tuen Mun, Hong Kong, China; Department of Sociology and Social Policy, Lingnan University, 8 Castle Peak Road, Tuen Mun, Hong Kong, China

**Keywords:** Age discrimination, Life satisfaction, Happiness, World Values Survey

## Abstract

**Objectives:**

Ageism against older adults is a global concern with significant implications for health and well-being. While the individual-level consequences of ageism are well-documented, the moderating role of structural sociopolitical factors remains underexplored. This study addresses these gaps by examining the relationship between perceived ageism and subjective well-being among older adults and investigating how macro-level sociopolitical conditions (e.g., economic conditions, political systems, cultural values) moderate these effects.

**Methods:**

: We utilized data from the World Values Survey (Wave 6), comprising 15,697 older adults (aged 55+) across 43 countries. Perceived ageism was measured by negative age-related stereotypes and perceived social status devaluation. Subjective well-being was assessed using life satisfaction and happiness. Macro-level sociopolitical moderators included gross domestic product (GDP) per capita, political stability, individualism, and long-term orientation.

**Results:**

: Perceived ageism was negatively associated with both life satisfaction and happiness, after adjusting for sociodemographic variables and macro-level sociopolitical factors. Higher GDP per capita mitigated these adverse effects, suggesting that economic prosperity buffers against the negative impact of ageism. Conversely, political stability unexpectedly amplified the detrimental effects of ageism on subjective well-being. Additionally, long-term cultural orientation strengthened the negative association between age-related stereotypes and well-being, while individualism did not exhibit a significant moderating effect.

**Discussion:**

: These findings highlight the crucial role of structural conditions in shaping the well-being outcomes of ageism. By demonstrating how economic and political contexts influence the consequences of ageism, this study provides valuable insights for policymakers aiming to develop targeted interventions and policies that promote the well-being of older populations.

The global population of older adults is growing at an unprecedented rate. As of 2024, approximately 1.2 billion people worldwide are aged 60 or above—a figure projected to double to around 2.1 billion by 2050 (United Nations, 2024). Despite this demographic shift, negative attitudes toward older individuals persist. Ageism—defined as the stereotypes, prejudice, and discrimination based on age—has been widely documented to undermine older adults’ well-being (Chang et al., 2020; Kang & Kim, 2022). Globally, nearly one in two people hold ageist views that portray older adults as frail or incompetent, a bias that pervades workplaces, healthcare, social services, and media (World Health Organization [WHO], 2021). Empirical research consistently links perceived age-based discrimination to lower life satisfaction (Avidor et al., 2017; Kim & Kang, 2017), reduced happiness (Jung & Kim, 2023), higher depression (Ayalon, 2018; Lyons et al., 2018), and increased loneliness (Mikton et al., 2021; Shiovitz-Ezra et al., 2018), independent of objective socioeconomic conditions.

While research has extensively documented the harms of ageism, much of the literature has focused on micro-level mechanisms—such as individual differences in age, gender, subjective life expectancy, and personal control—that may moderate the impact of perceived age discrimination on mental well-being (Avidor et al., 2017; Yuan, 2007). Little attention has been paid to the role of macro-level systems in shaping societal attitudes and their psychological consequences (Chang et al., 2020). The Socio-Ecological Model (Bronfenbrenner, 1979) posits that individual well-being results from dynamic interactions across multiple layers of influence, from immediate relationships to national policies and cultural norms. Macro-level factors, including economic development, governance stability, and cultural values, may influence how perceived ageism affects older adults’ well-being by shaping disparities in access to resources, social protections, and collective attitudes toward aging. Furthermore, over 80% of aging studies originate in high-income countries, which account for only 31% of the global population aged 65 and older (Hyde, 2024), leaving the experiences of older adults in low- and middle-income countries understudied.

This study addresses these gaps by examining the relationship between perceived ageism and the subjective well-being of older adults, with a focus on how macro-level factors—such as economic conditions, political governance, and cultural orientation—moderate this relationship. Drawing on cross-national data from the World Values Survey across 43 societies, we employ multilevel modeling to disentangle the interplay between individual perceptions of ageism and broader societal contexts. By extending aging research beyond micro-level analyses, our approach demonstrates how social systems shape the health consequences of ageism. The findings can inform the design of context-specific interventions aimed at mitigating the negative effects of ageism and promoting healthy aging across diverse cultural and economic settings.

## Perceived ageism and subjective well-being among older adults

Ageism refers to stereotypes (thoughts), prejudice (feelings), and discrimination (behaviors) directed towards people based on their age (WHO, 2021). According to the WHO’s Global Report on Ageism (2021), one in two people worldwide holds ageist attitudes toward older adults, though prevalence varies significantly across different cultural and socioeconomic settings. Perceived ageism is a well-documented risk factor for lower subjective well-being among older adults, including lower life satisfaction (Avidor et al., 2017; Kim & Kang, 2017), diminished happiness (Jung & Kim, 2023), higher depression (Ayalon, 2018; Lyons et al., 2018), and heightened loneliness (Mikton et al., 2021; Shiovitz-Ezra et al., 2018).

According to Stereotype Embodiment Theory (Levy, 2009), the detrimental effects of ageism on well-being occur via three interrelated pathways. Psychologically, when older adults internalize negative age stereotypes, their self-efficacy declines and emotional distress increases, which contributes to depression and chronic stress (Levy & Leifheit-Limson, 2009; Wurm & Benyamini, 2014). Behaviorally, these internalized beliefs lead individuals to adopt fatalistic attitudes toward aging, reducing their engagement in preventive health behaviors such as regular medical screenings or physical exercise (Chang et al., 2020; Levy & Myers, 2004). Physiologically, the chronic stress associated with perceived discrimination results in heightened cardiovascular reactivity and inflammation, processes that over time can precipitate serious health conditions like hypertension and cardiovascular disease (Allen, 2016; Levy et al., 2000).

Beyond individual-level processes, the institutional pathway further compounds the negative impact of ageism. Ageist practices embedded within social structures can disrupt social relationships and restrict access to quality healthcare services, thereby reinforcing marginalization. Older adults who face discrimination often withdraw from social interactions to avoid rejection, resulting in isolation and diminished social support (Voss et al., 2018). Institutional ageism manifests through systemic barriers such as age-based treatment rationing and exclusion from clinical trials, limiting opportunities for adequate care and perpetuating a cycle of self-directed ageism (Chang et al., 2020). Given the far-reaching health consequences of ageism, combating ageism has been designated as a key action area in the United Nations Decade of Healthy Ageing (2021–2030) (United Nations, 2020).

## Perceived ageism, macro-level sociopolitical factors, and well-being

The relationship between perceived ageism and subjective well-being among older adults is likely shaped by macro-level factors, as socio-ecological and cultural factors are essential contexts for human thought and behavior (Bronfenbrenner, 1979). Economic, political, and cultural conditions not only define the socio-ecological landscape in which aging occurs but also determine access to resources and the societal roles assigned to older adults. By exploring how gross domestic product (GDP) per capita, governance stability, and cultural orientations moderate the effects of perceived ageism, we can gain deeper insights into the complex interplay between societal context and individual experience.

### Economic development

Economic development may moderate the relationship between perceived ageism and well-being among older adults by shaping societal attitudes, social protections, and access to resources. Modernization theory suggests that while early industrialization tends to devalue older adults by prioritizing youth-centric productivity, perceptions about older people’s status and competence increase in more advanced stages of modernization (Palmore & Manton, 1974). For instance, wealthier European nations typically report warmer societal attitudes and higher subjective social status among older adults (Swift et al., 2014; Vauclair et al., 2015). In high-GDP countries, robust social safety nets—such as comprehensive pensions and healthcare—mitigate the harmful effects of ageism by fulfilling older adults’ needs for autonomy and security (Aboderin, 2010). Thus, older individuals’ success in maintaining their well-being despite potential stressors appears to be limited to countries with high economic development (Steptoe et al., 2015).

Conversely, in low- and middle-income countries, where economic constraints limit welfare provisions, the negative psychological effects of ageism may be amplified. Chang et al.’s (2020) systematic review found a higher prevalence of significant ageism-health associations in less-developed countries. With weaker healthcare systems, fewer social protections, and heightened intergenerational competition for scarce resources, older adults in low- and middle-income countries face greater vulnerability to discrimination and social exclusion (North & Fiske, 2015). The lack of adequate mental health support further exacerbates these challenges, as illustrated by stark disparities in mental health service availability between high- and low-income countries (WHO, 2020). In such contexts, even positive societal stereotypes about older adults’ competence can backfire, as economic limitations create a gap between expectations and opportunities, fostering frustration and decreased well-being (Fasel et al., 2021). These disparities underscore the critical role of economic development in shaping how perceived ageism translates into well-being outcomes for older populations worldwide.

### Political stability

Political stability, characterized by consistent governance and the absence of systemic violence or upheaval, may shape how perceived ageism affects the subjective well-being of older adults. Welfare State Theory (Esping-Andersen, 1990) posits that stable governance enables long-term investments in social protections—such as pensions, universal healthcare, and anti-discrimination policies—that safeguard older adults’ autonomy and dignity. These systems act as buffers, mitigating the psychological and material harms of ageism. Conversely, political instability may foster policy myopia, where governments prioritize short-term spending over elder welfare (Darby et al., 2004). For instance, in conflict-affected regions like Syria or Afghanistan, political unrest depletes healthcare infrastructure and redirects funds to military spending, leaving older adults without critical mental health support (Ghobarah et al., 2004). Irregular power transfers and civil wars further reduce economic growth, shrinking resources for elder care and exacerbating vulnerabilities (Przeworski et al., 2000). In such contexts, ageism intersects with a lack of safety nets, further reducing the well-being of older adults.

Moreover, political instability can erode social capital—the networks, trust, and community cohesion that are vital for buffering against ageism (Meyer, 2017). Research indicates that older adults often experience fewer supportive social ties during crises, which compromises their ability to access financial or emotional assistance during disasters (Cassar et al., 2011). Political instability further disrupts public spaces and programs, such as senior centers, facilitating social engagement and mitigate isolation (Mollica et al., 2004). In contrast, stable societies foster environments where collective support and strong community ties thrive. For instance, in the politically stable Nordic countries, community initiatives and volunteer programs for older adults significantly reduce feelings of loneliness and stigma (Klinenberg, 2018). Additionally, effective governance and high levels of trust in public institutions are closely associated with increased life satisfaction among older adults (Ma et al., 2024). Consequently, in politically unstable contexts where social capital is eroded and public trust is diminished, the detrimental effects of perceived ageism, including reduced self-esteem and increased stress, are likely to be more pronounced.

### Cultural values

We examine two cultural values—individualism and long-term orientation—as moderators of the relationship between perceived ageism and well-being in older adults. These values are selected for their distinct relevance to ageism dynamics and empirically linked to societal attitudes toward aging (Ng & Lim-Soh, 2021; North & Fiske, 2015).

Individualism refers to a cultural emphasis on personal autonomy, self-reliance, and individual achievement, which may shape how older adults perceive and respond to age-related stereotypes and discrimination. Individualistic societies tend to hold more positive attitudes toward older adults compared to collectivist cultures. North and Fiske’s (2015) meta-analysis found that collectivist cultural norms correlate with resentment toward older adults, who may be perceived as unfairly benefiting from societal support without adequately contributing, particularly in domains like labor and technology. Studies also report higher rates of perceived age discrimination in Eastern/collectivist contexts (Löckenhoff et al., 2009). In contrast, individualism emphasizes personal autonomy, self-reliance, and achievement (Ho & Chiu, 1994), which may buffer against the negative effects of ageism. For example, a sense of control—central to individualistic values—has been shown to mitigate the mental health consequences of perceived age discrimination (Yuan, 2007). Older adults who embrace individualistic values are better equipped to resist negative stereotypes, as personal resources like self-esteem mitigate the psychological distress linked to ageism (Bergman, 2022). By prioritizing self-defined identity over societal judgments, individualism may reduce the harmful impact of ageism on well-being.

Long-term orientation is “the extent to which a culture programs its members to accept delayed gratification of their material, social, and emotional needs.” (Hofstede, 2001, p. xix). Though long-term orientation is theorized to promote respect for tradition and older people (Hofstede, 2001), empirical evidence shows societies high in long-term orientation exhibit greater implicit and explicit age bias, colder attitudes toward older adults, and more negative aging narratives (Ackerman & Chopik, 2021; Ng & Lim-Soh, 2021). This paradox may stem from economic rationality of long-term orientation: older adults are devalued as “low-potential” investments compared to younger populations, who are prioritized for their perceived role in driving innovation and long-term growth (Aurigemma & Mattson, 2019; Bukowski & Rudnicki, 2019). In aging societies, this calculus intensifies intergenerational tensions, framing older adults as burdens on healthcare and economic resources (Ng et al., 2021). For instance, countries with high long-term orientation, like Japan and South Korea, implement youth-centric workplace policies and marginalize older workers. While short-term-oriented cultures often implement stronger elder-support policies (e.g., pensions, anti-discrimination laws), the focus of long-term orientation on future rewards (e.g., security, innovation) clashes with the need to address older adults’ immediate concerns, a tension that fuels resentment toward older adults.

## The present study

This study examines the relationship between perceived ageism and subjective well-being among older adults while also assessing the moderating effects of macro-level sociopolitical conditions. Building on prior research that has documented the deleterious effects of ageist attitudes on health and quality of life (Levy, 2009; North & Fiske, 2015), we investigate two distinct indicators of ageism: negative age stereotypes and perceived devaluation of social status. Age-related stereotypes assess societal perceptions of older adults’ competence and warmth, directly reflecting prejudicial attitudes. Perceived age-based status devaluation, meanwhile, measures the extent to which older individuals are viewed as occupying a lower societal standing—a structural dimension linked to experiences of marginalization and discrimination. Research has shown that perceptions of low social status predict exposure to discrimination (Adler et al., 2000; Vauclair et al., 2016). Cross-cultural surveys like the European Social Survey similarly incorporate perceived social status as an ageism indicator (Garstka et al., 2004). Thus, the two variables capture both the cognitive and structural dimensions of ageism.

In addition, we explore how broader societal contexts—specifically economic development, stability of governance, long-term vs. short-term cultural orientation, and individualism vs. collectivism—moderate the impact of perceived ageism. Data for this investigation were drawn from Wave 6 of the World Values Survey (WVS6), focusing on participants aged 55 and over across 43 societies. This study aims to answer several key questions: (1) How do negative age stereotypes and perceived social devaluation affect subjective well-being among older adults? (2) In what ways do macro-level conditions such as higher economic development, stronger political stability, and particular cultural orientations buffer or exacerbate the negative effects of ageism on well-being?

Based on the literature, we develop the following hypotheses:

H1: Higher levels of perceived ageism are negatively associated with subjective well-being among older adults.

H2: Economic development attenuates the negative association between ageism and subjective well-being.

H3: Governance quality weakens the negative effects of ageism on well-being.

H4: Long-term cultural orientation strengthens the relationship between perceived ageism and subjective well-being.

H5: Individualism weakens the negative effects of ageism on subjective well-being.

## Methods

### Data

This study utilizes data from WVS6, conducted between 2010 and 2014 across 60 countries and territories. As the only large-scale, globally representative survey to explicitly measure ageism, WVS6 is uniquely suited for analyzing the cross-cultural impact of perceived ageism on well-being. To focus on older adults, the sample was restricted to individuals aged 55 and above.

Three society-level variables were incorporated from external sources. First, economic conditions were proxied by GDP per capita (constant 2015 US$) from the World Bank. Second, political stability was measured using the Political Stability and Absence of Violence/Terrorism index from the Worldwide Governance Indicators. For both, data from each society’s WVS6 survey year were used, as economic and governance conditions fluctuate over time. Third, two cultural dimensions—long-term orientation and Individualism—were derived from Hofstede’s index. The most recent available Hofstede scores were used, as cultural values exhibit relative stability over time.

Countries with incomplete data on these four societal-level variables were excluded from the analysis. India was also omitted, as it did not administer the ageism module in WVS6. Using listwise deletion to handle missing data produced an analytical sample of 15,697 older adults from 43 societies. Results obtained through multiple imputations for missing values were consistent with those from the listwise deletion approach (available upon request); therefore, we report only the listwise deletion results for simplicity.

### Measures

#### Dependent variable: subjective well-being

Subjective well-being refers to individuals’ overall evaluations of their lives, capturing both cognitive and affective dimensions (Diener et al., 2002). In this study, subjective well-being was measured using two indicators: life satisfaction (cognitive component) and happiness (affective component), offering a nuanced understanding of distinct dimensions of well-being across societies.

*Life satisfaction* is measured with a single item asking respondents, “All things considered, how satisfied are you with your life as a whole these days?” using a 10-point scale from 1 (“completely dissatisfied”) to 10 (“completely satisfied”). *Happiness* is gauged with a question that asks respondents to rate their overall happiness on a 4-point scale ranging from 1 (“not at all happy”) to 4 (“very happy”).

#### Independent variable: perceived ageism

Perceived ageism is operationalized through two components: negative age-related stereotypes and perceived age-based status devaluation. *Negative age stereotypes* were measured by averaging responses to three items that asked how likely respondents believed most people in their country viewed individuals over 70 as friendly, competent, and respected. Responses were recorded on a 1–5 scale and reverse-coded so that higher scores indicate stronger negative stereotypes.

*Perceived age-based status devaluation* was assessed by asking respondents to indicate where most people would place the social position of those over 70 on a 1–10 social ladder. The variable was coded so that higher scores reflected a lower perceived status.

#### Societal-level moderators

*Economic development* was measured using GDP per capita (constant 2015 US$) from World Bank national accounts data and Organization for Economic Co-operation and Development (OECD) National Accounts data files. This indicator captures the overall economic performance and average material living standard within a country. In this study, GDP per capita was log-transformed to correct for skewness and ensure a more linear relationship in the analysis.

*Stability of governance* was measured using the Political Stability and Absence of Violence/Terrorism index from Worldwide Governance Indicators. This index, ranging from approximately −2.5 to 2.5, aggregates data from multiple sources to assess the likelihood of political instability and politically motivated violence, including terrorism, within a society. Higher values indicate better political stability.

Cultural values were assessed using two of Hofstede’s cultural dimensions: long-term orientation and individualism. *Long-term orientation* reflects the degree to which societies prioritize tradition over adapting to future challenges. Societies with lower long-term orientation scores emphasize maintaining established customs and resisting rapid social changes. *Individualism* measures the degree of independence among individuals in society. In societies with lower individualism, individuals are more dependent on their social groups. This study used scores obtained from Hofstede Insights, last updated in October 2023. Both cultural dimensions exhibited substantial variation across societies, with scores ranging from 0 to 100.

#### Control variables

We controlled for several sociodemographic variables, including *age* (in years), *sex* (male vs. female), *marital status* (single, married/cohabiting, and divorced/widowed/separated), and *parental status* (no children vs. having children). To account for socioeconomic background, *education* was grouped into lower than secondary, secondary, tertiary or above, and unknown. *Income* was measured using an 11-point ladder, with higher values indicating higher income status.

### Analytic strategy

All analyses were performed using Stata v18. Descriptive statistics were used to summarize the characteristics of the population (see Table 1 and Supplementary Table 1). The zero-order Pearson correlations for all key individual-level variables are presented in Supplementary Table 2. Multilevel Linear Regressions were conducted to examine the main and interactive effects of perceived ageism and macro-level sociopolitical factors on subjective well-being (life satisfaction and happiness). First, we estimated unconditional (null) models with no explanatory variables and calculated the Intraclass Correlation Coefficient (ICC) to determine the proportion of variance attributable to the country level. Then subjective well-being outcomes were regressed on two indicators of perceived ageism and four sociopolitical factors, controlling for sociodemographic covariates. Next, two-way interaction terms between perceived ageism indicators and sociopolitical variables were computed to test whether structural factors moderated the relationship between perceived ageism and subjective well-being. Simple slope tests for significant interactions were conducted using predicted values of the dependent variables, with moderator scores set one standard deviation above and below the mean (Supplementary Figure 1). Variance inflation factors (VIFs) of the independent variables were estimated to assess multicollinearity. All VIF values were below 2, indicating that multicollinearity was not a significant concern. Unstandardized coefficients with 95% confidence intervals were reported. A *p*-value of .05 was set as the threshold for statistical significance.

## Results

### Main effect of perceived ageism and sociopolitical factors on subjective well-being

We first estimated unconditional null models without any explanatory variables (Model 0a in Table 2 and Model 0b in Table 3). The ICC was 0.160 for life satisfaction and 0.164 for happiness. This indicates that country-level differences account for approximately 16% of the variance in each outcome, justifying our multilevel approach (Hox, 2010).

**Table 1.** Descriptive statistics (*N *= 15,697).

Variable	Mean (SD)	Min	Max	*N* (%)
**Life satisfaction**	6.80 (2.32)	1	10	
**Happiness**	3.04 (0.76)	1	4	
**Age stereotype**	2.27 (0.91)	1	5	
**Age-based status devaluation**	5.81 (2.62)	1	10	
**GDP per capita (logged)**	9.47 (1.02)	7.18	10.92	
**Stability of governance**	0.08 (0.90)	−2.68	1.37	
**Long-term orientation**	48.24 (23.22)	1	100	
**Individualism**	49.47 (23.96)	0	100	
**Age**	65.42 (7.88)	55	99	
**Gender**				
** Male**				7,376 (46.99)
** Female**				8,321 (53.01)
**Marital status**				
** Single**				779 (4.96)
** Married/cohabited**				10,547 (67.19)
**Divorced/widowed/separated**				4,371 (27.85)
**Parental status**				
** No children**				1,657 (10.56)
** Has children**				14,040 (89.44)
**Income level**	4.66 (2.36)	1	11	
**Education**				
**Lower than secondary school**				6,165 (39.28)
** Secondary school**				5,923 (37.73)
**Tertiary education or above**				3,293 (20.98)
** Unknown**				316 (2.01)

**Table 2.** Perceived ageism, macro-level sociopolitical factors, and life satisfaction among older adults.

Variable	Model 0a		Model 1a		Model 2a		Model 3a	
	*B* [95% CI]	*p*	*B* [95% CI]	*p*	*B* [95% CI]	*p*	*B* [95% CI]	*p*
**Intercept**	6.723 [6.44, 7.00]	<.001	−0.217 [−3.918, 3.485]	.909	1.788 [−2.150, 5.726]	.374	1.510 [−2.368, 5.388]	.445
**Negative age stereotype**			−0.226 [−0.264, −0.187]	<.001	−1.093 [−1.648, −0.538]	<.001	−0.225 [−0.264, −0.187]	<.001
**Age-based status devaluation**			−0.052 [−0.066, −0.038]	<.001	−0.053 [−0.066, −0.039]	<.001	−0.339 [−0.543, −0.135]	.001
**GDP per capita (logged)**			0.682 [0.227, 1.137]	.003	0.443 [−0.041, 0.927]	.073	0.470 [−0.006, 0.947]	.053
**Stability of governance**			−0.163 [−0.533, 0.206]	.386	0.05 [−0.351, 0.450]	.808	−0.009 [−0.400, 0.381]	.964
**Long-term orientation**			−0.010 [−0.022, 0.001]	.085	−0.005 [−0.018, 0.007]	.395	−0.007 [−0.020, 0.005]	.242
**Individualism**			−0.000 [−0.019, 0.019]	.990	−0.000 [−0.020, 0.020]	.995	0.003 [−0.017, 0.022]	.792
**Negative age stereotype**								
**× GDP per capita (logged)**					0.102 [0.035, 0.170]	.003		
**× Stability of governance**					−0.095 [−0.164, −0.027]	.006		
**× Long-term orientation**					−0.002 [−0.004, −0.000]	.036		
**× Individualism**					0.000 [−0.003, 0.003]	.936		
**Age-based status devaluation**								
**× GDP per capita (logged)**							0.035 [0.010, 0.059]	.005
**× Stability of governance**							−0.029 [−0.054, −0.004]	.023
**× Long-term orientation**							−0.000 [−0.001, 0.000]	.159
**× Individualism**							−0.000 [−0.001, 0.001]	.483
**Age**			0.009 [0.005, 0.014]	<.001	0.009 [0.005, 0.014]	<.001	0.009 [0.005, 0.014]	<.001
**Female**			0.176 [0.109, 0.243]	<.001	0.175 [0.108, 0.243]	<.001	0.177 [0.109, 0.244]	<.001
**Marital status (ref: single)**								
**Married/cohabited**			0.338 [0.166, 0.510]	<.001	0.340 [0.168, 0.512]	<.001	0.342 [0.170, 0.514]	<.001
**Divorced/widowed/separated**			−0.195 [−0.372, −0.017]	.032	−0.193 [−0.370, −0.015]	.033	−0.194 [−0.372, −0.017]	.032
**Parental status (ref: no children)**								
**Has children**			0.146 [0.024, 0.269]	.019	0.148 [0.025, 0.270]	.018	0.144 [0.022, 0.267]	.021
**Income level**			0.179 [0.164, 0.194]	<.001	0.179 [0.164, 0.193]	<.001	0.179 [0.164, 0.193]	<.001
**Education (ref: lower than secondary school)**								
**Secondary school**			0.126 [0.042, 0.210]	.003	0.128 [0.043, 0.212]	.003	0.129 [0.045, 0.214]	.003
**Tertiary education or above**			0.222 [0.121, 0.323]	<.001	0.223 [0.122, 0.325]	<.001	0.224 [0.123, 0.326]	<.001
**Unknown**			0.109 [−0.165, 0.384]	.435	0.116 [−0.158, 0.391]	.406	0.120 [−0.154, 0.395]	.390
**Variance (Level 2)**	0.859 [0.558, 1.320]		0.554 [0.358, 0.856]		0.560 [0.362, 0.866]		0.549 [0.355, 0.849]	
**Variance (Level 1)**	4.522 [4.423, 4.623]		4.175 [4.083, 4.268]		4.170 [4.079, 4.264]		4.172 [4.080, 4.265]	
**Intraclass correlation**	0.160		0.117		0.118		0.116	
**Log Likelihood**	−34,203.8		−33,569.7		−33,561.8		−33,563.9	

*Note*. ref = reference.

**Table 3.** Perceived ageism, macro-level sociopolitical factors, and happiness among older adults.

Variable	Model 0a		Model 1a		Model 2a		Model 3a	
	*B* [95% CI]	*p*	*B* [95% CI]	*p*	*B* [95% CI]	*p*	*B* [95% CI]	*p*
**Intercept**	3.032 [2.939, 3.124]	<.001	1.369 [0.065, 2.673]	.040	1.656 [0.281, 3.031]	.018	1.341 [−0.032, 2.715]	.056
**Negative age stereotype**			−0.047 [−0.060, −0.034]	<.001	−0.171 [−0.354, 0.011]	.066	−0.046 [−0.059, −0.034]	<.001
**Age-based status devaluation**			−0.012 [−0.017, −0.008]	<.001	−0.012 [−0.017, −0.008]	<.001	−0.006 [−0.073, 0.061]	.869
**GDP per capita (logged)**			0.166 [0.006, 0.327]	.042	0.129 [−0.040, 0.298]	.135	0.169 [0.000, 0.338]	.049
**Stability of governance**			0.068 [−0.062, 0.198]	.307	0.121 [−0.019, 0.260]	.089	0.106 [−0.032, 0.244]	.133
**Long-term orientation**			−0.001 [−0.005, 0.003]	.609	−0.001 [−0.005, 0.004]	.770	−0.001 [−0.006, 0.003]	.595
**Individualism**			−0.006 [−0.012, 0.001]	.086	−0.005 [−0.012, 0.002]	.168	−0.006 [−0.013, 0.001]	.117
**Negative age stereotype**								
** × GDP per capita (logged)**					0.016 [−0.006, 0.038]	.154		
** × Stability of governance**					−0.024 [−0.046, −0.001]	.038		
** × Long-term orientation**					−0.000 [−0.001, 0.000]	.612		
** × Individualism**					−0.000 [−0.001, 0.001]	.453		
**Age-based status devaluation**								
** × GDP per capita (logged)**							−0.001 [−0.009, 0.007]	.865
** × Stability of governance**							−0.008 [−0.016, 0.000]	.060
** × Long-term orientation**							0.000 [−0.000, 0.000]	.897
** × Individualism**							−0.000 [−0.000, 0.000]	.924
**Age**			0.003 [0.001, 0.004]	<.001	0.003 [0.001, 0.004]	<.001	0.003 [0.001, 0.004]	<.001
**Female**			0.063 [0.041, 0.086]	<.001	0.064 [0.041, 0.086]	<.001	0.064 [0.042, 0.086]	<.001
**Marital status (ref: single)**								
** Married/cohabited**			0.147 [0.090, 0.204]	<.001	0.147 [0.091, 0.204]	<.001	0.148 [0.091, 0.205]	<.001
** Divorced/widowed/separated**			−0.074 [−0.133, −0.016]	.013	−0.073 [−0.132, −0.015]	.014	−0.073 [−0.131, −0.014]	.015
**Parental status (ref: no children)**								
** Has children**			0.120 [0.079, 0.160]	<.001	0.12 [0.080, 0.161]	<.001	0.118 [0.078, 0.158]	<.001
**Income level**			0.039 [0.034, 0.044]	<.001	0.039 [0.034, 0.044]	<.001	0.039 [0.034, 0.044]	<.001
**Education (ref: lower than secondary school)**								
** Secondary school**			0.033 [0.005, 0.061]	.021	0.034 [0.006, 0.061]	.018	0.034 [0.006, 0.062]	.016
** Tertiary education or above**			0.082 [0.049, 0.115]	<.001	0.084 [0.051, 0.117]	<.001	0.085 [0.051, 0.118]	<.001
** Unknown**			0.020 [−0.070, 0.111]	.661	0.024 [−0.067, 0.114]	.609	0.018 [−0.072, 0.109]	.692
**Variance (Level 2)**	0.094 [0.061, 0.145]		0.069 [0.045, 0.107]		0.069 [0.045, 0.107]		0.070 [0.046, 0.108]	
**Variance (Level 1)**	0.479 [0.469, 0.490]		0.453 [0.443, 0.463]		0.453 [0.443, 0.463]		0.452 [0.443, 0.463]	
**Intraclass correlation**	0.164		0.132		0.133		0.134	
**Log Likelihood**	−16,591.2		−16,138.6		−16,133.6		−16,132.3	

*Note*. ref = reference.

Table 2 and Table 3 present the main and interactive effects of perceived ageism and sociopolitical factors on life satisfaction and happiness, respectively. Results from multilevel analysis show that stronger negative age-related stereotypes were associated with lower life satisfaction (*b* = −0.226, 95% CI = [−0.264, −0.187], *p* < .001). Similarly, greater status devaluation was linked to reduced life satisfaction (*b* = −0.052, 95% CI = [−0.066, −0.038], *p* < .001). Consistent with these findings, negative age-related stereotypes (*b* = −0.047, 95% CI = [−0.060, −0.034], *p* < .001) and greater status devaluation (*b* = −0.012, 95% CI = [−0.017, −0.008], *p* < .001) were also negatively associated with happiness. These results support Hypothesis 1, which posits that perceived ageism is linked to lower subjective well-being among older adults.

Regarding macro-level factors, GDP per capita was the only significant predictor of both life satisfaction (*b* = 0.682, 95% CI = [0.227, 1.137], *p* = .003) and happiness (*b* = 0.166, 95% CI = [0.006, 0.327], *p* = .042), indicating that older adults in wealthier societies tend to report higher subjective well-being. Additionally, long-term orientation showed a negative association with life satisfaction (*b* = −0.010, 95% CI [−0.022, 0.001], *p* = .085), though this effect did not meet conventional thresholds for statistical significance. Moreover, individualism was negatively related to happiness (*b* = −0.006, 95% CI [−0.012, 0.001], *p* = .086), but this relationship fell short of statistical significance as well.

### Interactive effect of perceived ageism and sociopolitical factors on subjective well-being

The interaction between age-related stereotypes and GDP per capita (*b* = 0.102, 95% CI = [0.035, 0.170], *p* = .003), as well as between perceived status devaluation of older people and GDP per capita (*b* = 0.035, 95% CI = [0.010, 0.059], *p* = .005) were negative for life satisfaction, suggesting economic prosperity buffers against ageism (Supplementary Figure 1a and b). The findings support Hypothesis 2, indicating that older adults living in wealthier societies were less vulnerable to the negative effects of perceived ageism on their subjective well-being.

Contrary to Hypothesis 3, political stability exacerbated the negative impact of perceived ageism. The detrimental effects of age-related stereotypes (*b* = −0.095, 95% CI = [−0.164, −0.027], *p* = .006) and perceived social position (*b* = −0.029, 95% CI = [−0.054, −0.004], *p* = .023) on life satisfaction were stronger in more politically stable societies (Supplementary Figure 1c and d). These results suggest that political stability may intensify the negative effects of perceived ageism.

Long-term orientation significantly strengthened the negative relationship between age-related stereotypes and life satisfaction (*b* = −0.002, 95% CI [−0.004, −0.000], *p* = .036; Supplementary Figure 1e). However, it did not moderate the relationship between perceived social position and life satisfaction, nor were there significant moderating effects of individualism. These results were inconsistent with Hypothesis 4.

However, different patterns emerged for happiness, as shown in Table 3. Among the four macro-level factors, only political stability remained a significant moderator. The negative effects of age-related stereotypes on happiness were more pronounced in politically stable societies (*b* = −0.024, 95% CI = [−0.046, −0.001], *p* = .038; Supplementary Figure 1f). Similarly, the relationship between perceived status devaluation and political stability was negative but did not reach conventional levels of statistical significance (*b* = −0.008, 95% CI = [−0.016, 0.000], *p* = .060). These results further challenge Hypothesis 3, suggesting that political stability inadvertently reinforces negative age-related biases that undermine older adults’ well-being. Future research with larger samples is needed to confirm this potential effect.

### Robustness check

To ensure the stability of our findings, we conducted two sets of robustness checks (see Supplementary Methods for full details and tables). First, we replicated our analyses on subsamples with stricter age criteria (55–85 and 55–80). Second, we re-estimated the models using outcome variables adjusted for outliers. Across all tests, the direction, magnitude, and significance of the main and interactive effects remained consistent with our primary models. This confirms that our results, including the unexpected findings, are robust and not dependent on the inclusion of the oldest-old or the influence of extreme values.

## Discussion and conclusion

This study is among the first to examine the main and interactive effects of perceived ageism and macro-level sociopolitical factors on the subjective well-being of older adults. Our analysis of data from 15,697 older individuals across 43 societies reveals that perceived ageism—captured by negative age-related stereotypes and status devaluation—is consistently associated with lower life satisfaction and happiness. Notably, economic prosperity buffered the negative effects of ageism on life satisfaction. However, political stability unexpectedly amplified the negative impact of ageism on both life satisfaction and happiness, challenging the assumption that stable governance inherently protects marginalized groups. Among cultural values, long-term orientation exacerbated the detrimental effects of negative age-related stereotypes on life satisfaction, while individualism did not significantly moderate these relationships. These findings advance our understanding of how structural and cultural contexts interact with individual experiences of ageism to influence subjective well-being.

These findings extend the existing literature by demonstrating that the consequences of perceived ageism are not uniform across societies but are instead shaped by broader sociopolitical and cultural contexts. The detrimental effects of negative ageist stereotypes on both evaluative (life satisfaction) and affective (happiness) dimensions of well-being align with prior empirical studies documenting the harmful impact of ageism (Chang et al., 2020; Kim & Kang, 2017). Negative stereotypes can erode self-worth and intensify feelings of social exclusion, thereby reducing life satisfaction and happiness (Levy, 2009). Additionally, the perceived devaluation of older adults’ social status is negatively associated with well-being, consistent with previous research (Demakakos et al., 2008; Hu et al., 2005). Social identity theory suggests that occupying a lower-status position fosters identity-based stress, which can lower self-esteem and psychological well-being (Tajfel & Turner, 1986). Furthermore, perceptions of one’s place in the social hierarchy are linked to negative emotions, such as shame, and may affect health through neuroendocrine mechanisms (Wilkinson, 1996).

Crucially, by contextualizing these mechanisms within macro-level systems, this study shifts the focus from individual resilience to structural determinants, challenging assumptions that ageism’s consequences are invariant across societies. Our results show the critical role of macro-level economic conditions, as higher GDP per capita appears to mitigate the negative impacts of ageism. The buffering role of GDP per capital aligns with resource redistribution theories, which posit that economic prosperity enables robust social safety nets (e.g., pensions, healthcare) and thus reduces older adults’ dependency on familial or communal support and mitigates intergenerational resource competition (Aboderin, 2010). In wealthier societies, institutionalized ageism may be counterbalanced by policies that redistribute resources equitably, fostering environments where older adults feel valued rather than burdensome (Steptoe et al., 2015).

Our study reveals a counterintuitive finding: political stability exacerbates the negative impact of perceived ageism on well-being. It may be explained by the system justification theory (Jost & Banaji, 1994), as stable societies tend to legitimize existing social hierarchies, leading older adults to internalize ageist norms and experience heightened psychological distress. Governance stability may also foster complacency (Laurin et al., 2013), wherein marginalized groups rationalize inequalities rather than contest them, reinforcing acceptance of ageism as inevitable and deepening helplessness. Political stability may also institutionalize ageism under a “veil of legitimacy,” embedding discriminatory practices like mandatory retirement and healthcare rationing into policies that marginalize older adults (Santos et al., 2019). Older adults in these settings may face “benevolent ageism”, which includes paternalistic measures that impose dependency and social exclusion under the guise of protection (Cary et al., 2017). Thus, while political stability provides societal security, it concurrently entrenches discriminatory norms, stifling avenues for advocacy and perpetuating cycles of marginalization.

Similarly, the exacerbating effect of long-term orientation on the relationship between negative age stereotypes and life satisfaction underscores the influence of cultural values. Societies with a strong emphasis on long-term goals and future-oriented thinking may devalue older adults, who are often perceived as less future-oriented and less productive, thereby magnifying the negative effects of ageism (Hofstede, 2001). This dynamic is further reinforced by research on intergenerational resource tensions, which shows that older adults are often viewed as less contributory to economic growth and innovation, thereby justifying ageist practices under the guise of productivity and efficiency (North & Fiske, 2015). Consequently, the structural prioritization of youth in long-term orientation cultures amplifies the detrimental effects of negative age-related stereotypes on well-being, challenging the assumption that future-focused societies naturally support inclusive aging policies.

These findings carry significant policy implications. First, the detrimental impact of perceived ageism on subjective well-being underscores the urgent need for targeted interventions to reduce negative stereotypes and status devaluation of older adults. Public awareness campaigns and educational programs should challenge ageist attitudes and promote intergenerational solidarity—a goal embraced by the United Nations Decade of Healthy Aging (2021–2030) (United Nations, 2020). Second, the buffering effect of GDP per capita highlights the importance of investing in social welfare programs and infrastructure that support older populations. Policymakers should design economic strategies that foster growth while ensuring equitable access to resources, thereby enhancing older adults’ quality of life. Moreover, our findings caution against the unintended consequences of political stability and long-term cultural orientation, which may inadvertently reinforce entrenched ageist norms. In politically stable contexts, proactive measures such as participatory governance may help dismantle paternalistic policies by involving older adults in decision-making processes. Besides, efforts to promote intergenerational solidarity and challenge long-term cultural biases against older adults could help mitigate the adverse health outcomes. By anchoring these actions in combating ageism, policymakers can foster environments that prioritize older adults’ well-being and dignity, ensuring that healthy aging frameworks advance not only longevity but also equity and social inclusion across the life course.

The study has several limitations. First, cross-sectional data restrict our ability to establish causal relationships between perceived ageism and subjective well-being. Longitudinal research is needed to clarify the directionality and dynamics of these relationships. Second, our operationalization of ageism is limited to measures of negative age-related stereotypes and perceived age-based status devaluation. Although these dimensions capture important aspects of ageism, they do not encompass all its facets, such as direct experiences of discriminatory behavior, which may also be critical for understanding its full impact on well-being. Third, the reliance on self-reported measures may introduce biases related to cultural differences in response styles. Future research could incorporate objective indicators of well-being and sociopolitical conditions to validate and extend the present findings. Third, while this study examined four macro-level factors, other contextual variables, such as healthcare access, pension systems, and social policy frameworks, may further elucidate how sociopolitical environments shape older adults’ well-being. Moreover, our analysis uses country-level data, which cannot capture the cultural variability that may exist within nations. Since factors like cultural values can differ across sub-groups (e.g., indigenous communities), and our data cannot account for these granular differences, caution is warranted when generalizing from our findings. Lastly, the data used in this study were collected between 2010 and 2014. Since sociopolitical conditions have evolved, our findings may reflect the specific context of that period. Policy recommendations should therefore consider the dynamic nature of these conditions.

In conclusion, our study reveals that perceived ageism is a significant risk factor for reduced subjective well-being among older adults and that its impact is moderated by broader macro-level sociopolitical factors. Economic prosperity appears to offer a protective effect, whereas political stability and long-term orientation may inadvertently reinforce negative ageist attitudes. These findings not only enrich our theoretical understanding of ageism but also highlight the need for comprehensive, context-specific policy interventions to foster more inclusive and supportive environments for older adults.

## Supplementary Material

gbaf191_Supplementary_Data

## Data Availability

The data used in this study were obtained from the World Values Survey (WVS) Wave 6, which is publicly available (https://www.worldvaluessurvey.org).
