# Supplementary material for: Perceived ageism, macro-level sociopolitical factors, and subjective well-being: a cross-national study of older adults in 43 societies
Source: J Gerontol B Psychol Sci Soc Sci. 2025 Oct 6;80(12):gbaf191. doi: 10.1093/geronb/gbaf191 (PMC12605799; doi:10.1093/geronb/gbaf191)
Supplement: gbaf191_Supplementary_Data [file gbaf191_supplementary_data.zip › JGPS suppl Chen & Meng.docx]

***The Journals of Gerontology, Series B: Psychological Sciences and Social Sciences* Supplementary Material: Chen & Meng. Perceived ageism, macro-level sociopolitical factors, and subjective well-being: A cross-national study of older adults in 43 societies.**

**Supplementary Table 1.** List of Societies: Descriptive statistics by countries

| **Society** | ***n*** | **% of working**  **sample** | **GDP per capita**  **(logged)** | **Stability of**  **governance** | **Long-term**  **orientation** | **Individualism** | **Life satisfaction** | | **Happiness** | | **Age stereotype** | | **Age-based status devaluation** | |
| --- | --- | --- | --- | --- | --- | --- | --- | --- | --- | --- | --- | --- | --- | --- |
|  |  |  | **Mean** | **Mean** | **Mean** | **Mean** | **Mean** | **SD** | **Mean** | **SD** | **Mean** | **SD** | **Mean** | **SD** |
| Algeria | 146 | 0.93 | 8.33 | -1.19 | 25 | 29 | 6.95 | 2.15 | 3.01 | 0.63 | 2.53 | 1.21 | 4.92 | 2.61 |
| Argentina | 277 | 1.76 | 9.55 | 0.07 | 29 | 51 | 7.39 | 1.76 | 3.13 | 0.68 | 2.86 | 0.86 | 6.43 | 2.10 |
| Australia | 723 | 4.61 | 10.92 | 1.00 | 56 | 73 | 7.57 | 1.96 | 3.32 | 0.59 | 2.29 | 0.90 | 5.50 | 2.56 |
| Armenia | 344 | 2.19 | 8.02 | -0.06 | 38 | 17 | 4.56 | 2.51 | 2.80 | 0.88 | 2.39 | 1.08 | 6.77 | 2.62 |
| Brazil | 339 | 2.16 | 9.13 | -0.07 | 28 | 36 | 8.05 | 2.33 | 3.25 | 0.66 | 2.09 | 1.02 | 5.89 | 2.85 |
| Belarus | 375 | 2.39 | 8.69 | -0.09 | 53 | 48 | 5.46 | 2.18 | 2.58 | 0.72 | 2.20 | 0.80 | 6.34 | 2.52 |
| Chile | 274 | 1.75 | 9.47 | 0.33 | 12 | 49 | 7.03 | 2.03 | 2.97 | 0.71 | 2.55 | 0.85 | 6.14 | 2.24 |
| China | 477 | 3.04 | 8.86 | -0.54 | 77 | 43 | 6.95 | 2.15 | 3.02 | 0.57 | 2.06 | 0.72 | 5.06 | 1.99 |
| Colombia | 322 | 2.05 | 8.64 | -1.40 | 6 | 29 | 8.25 | 2.01 | 3.36 | 0.75 | 2.10 | 0.98 | 5.84 | 2.87 |
| Ecuador | 255 | 1.62 | 8.71 | -0.18 | 24 | 24 | 7.70 | 1.83 | 3.39 | 0.70 | 2.06 | 0.82 | 5.21 | 2.28 |
| Estonia | 576 | 3.67 | 9.66 | 0.61 | 71 | 62 | 5.79 | 2.14 | 2.68 | 0.66 | 2.57 | 0.91 | 7.44 | 2.03 |
| Georgia | 339 | 2.16 | 8.27 | -0.32 | 24 | 15 | 4.53 | 2.29 | 2.53 | 0.87 | 1.45 | 0.65 | 6.79 | 2.28 |
| Germany | 798 | 5.08 | 10.60 | 0.93 | 57 | 79 | 7.29 | 2.04 | 3.01 | 0.64 | 2.26 | 0.85 | 4.96 | 2.26 |
| Ghana | 102 | 0.65 | 7.40 | 0.13 | 1 | 9 | 6.27 | 2.38 | 3.32 | 0.79 | 1.99 | 1.00 | 4.60 | 2.62 |
| Iraq | 128 | 0.82 | 8.41 | -2.01 | 11 | 25 | 5.70 | 2.24 | 2.66 | 0.69 | 2.26 | 0.84 | 4.39 | 2.17 |
| Japan | 827 | 5.27 | 10.40 | 0.88 | 100 | 62 | 7.14 | 1.90 | 3.23 | 0.63 | 2.76 | 0.82 | 5.86 | 2.30 |
| Kazakhstan | 255 | 1.62 | 9.16 | -0.34 | 85 | 20 | 6.98 | 2.33 | 3.09 | 0.68 | 1.85 | 0.77 | 6.29 | 2.71 |
| Jordan | 223 | 1.42 | 8.38 | -0.54 | 20 | 20 | 6.47 | 2.33 | 2.95 | 0.73 | 2.06 | 0.90 | 4.17 | 2.28 |
| South Korea | 243 | 1.55 | 10.14 | 0.33 | 86 | 58 | 6.50 | 1.99 | 3.02 | 0.53 | 2.80 | 0.92 | 6.43 | 2.54 |
| Lebanon | 262 | 1.67 | 9.05 | -1.69 | 47 | 27 | 6.22 | 2.10 | 2.88 | 0.62 | 2.76 | 0.89 | 5.42 | 2.42 |
| Malaysia | 187 | 1.19 | 9.07 | -0.01 | 47 | 27 | 7.08 | 1.90 | 3.53 | 0.59 | 2.02 | 0.67 | 3.68 | 2.25 |
| Mexico | 268 | 1.71 | 9.20 | -0.66 | 23 | 34 | 8.52 | 2.23 | 3.47 | 0.74 | 1.93 | 0.94 | 4.48 | 3.34 |
| Morocco | 144 | 0.92 | 7.97 | -0.39 | 25 | 24 | 5.95 | 2.64 | 2.94 | 0.77 | 2.76 | 1.20 | 5.91 | 3.07 |
| Netherlands | 840 | 5.35 | 10.70 | 1.19 | 67 | 100 | 7.57 | 1.31 | 3.24 | 0.56 | 2.28 | 0.70 | 5.91 | 2.06 |
| Nigeria | 110 | 0.70 | 7.82 | -2.04 | 8 | 0 | 5.77 | 2.40 | 3.05 | 1.07 | 2.03 | 0.85 | 4.05 | 2.41 |
| Pakistan | 89 | 0.57 | 7.18 | -2.68 | 19 | 5 | 7.08 | 2.26 | 3.19 | 0.89 | 1.91 | 0.99 | 3.34 | 2.77 |
| Peru | 218 | 1.39 | 8.65 | -0.91 | 5 | 20 | 7.20 | 2.12 | 2.98 | 0.81 | 2.18 | 0.88 | 6.53 | 2.32 |
| Philippines | 274 | 1.75 | 7.86 | -1.19 | 46 | 17 | 7.01 | 3.02 | 3.28 | 0.73 | 2.21 | 0.93 | 5.32 | 3.11 |
| Poland | 335 | 2.13 | 9.35 | 1.05 | 49 | 47 | 6.78 | 2.10 | 3.11 | 0.58 | 2.40 | 0.93 | 6.49 | 2.63 |
| Romania | 539 | 3.43 | 9.02 | 0.08 | 32 | 46 | 6.32 | 2.54 | 2.59 | 0.77 | 2.00 | 0.84 | 6.82 | 2.96 |
| Russia | 725 | 4.62 | 9.12 | -1.00 | 58 | 46 | 5.71 | 2.35 | 2.75 | 0.72 | 2.20 | 0.90 | 7.58 | 2.26 |
| Singapore | 428 | 2.73 | 10.85 | 1.37 | 67 | 43 | 7.06 | 1.62 | 3.41 | 0.61 | 2.41 | 0.70 | 5.12 | 2.07 |
| Slovenia | 379 | 2.41 | 9.94 | 0.97 | 50 | 81 | 6.92 | 2.11 | 2.86 | 0.70 | 2.35 | 0.77 | 5.83 | 2.08 |
| South Africa | 413 | 2.63 | 8.74 | -0.05 | 18 | 23 | 6.61 | 2.31 | 3.10 | 0.87 | 1.94 | 0.87 | 4.40 | 2.32 |
| Spain | 355 | 2.26 | 10.14 | 0.02 | 47 | 67 | 6.65 | 1.79 | 2.95 | 0.55 | 2.57 | 0.98 | 6.17 | 2.21 |
| Sweden | 470 | 2.99 | 10.81 | 1.23 | 52 | 87 | 7.98 | 1.68 | 3.39 | 0.58 | 2.35 | 0.75 | 6.74 | 2.06 |
| Thailand | 255 | 1.62 | 8.62 | -1.31 | 67 | 19 | 7.53 | 1.95 | 3.30 | 0.66 | 1.65 | 0.59 | 4.16 | 2.41 |
| Tunisia | 188 | 1.20 | 8.27 | -0.90 | 24 | 27 | 5.32 | 2.51 | 2.80 | 0.65 | 2.02 | 1.04 | 4.57 | 2.98 |
| Turkey | 241 | 1.54 | 9.13 | -0.96 | 35 | 46 | 7.12 | 2.31 | 3.21 | 0.82 | 1.72 | 0.68 | 4.24 | 2.56 |
| Ukraine | 475 | 3.03 | 7.80 | -0.07 | 51 | 55 | 5.32 | 2.55 | 2.71 | 0.79 | 2.45 | 0.96 | 7.22 | 2.42 |
| Egypt | 302 | 1.92 | 8.10 | -1.64 | 22 | 13 | 5.45 | 2.79 | 1.89 | 0.94 | 1.95 | 0.90 | 5.25 | 2.92 |
| United States | 889 | 5.66 | 10.89 | 0.59 | 50 | 60 | 7.55 | 1.85 | 3.29 | 0.61 | 2.32 | 0.83 | 5.27 | 2.48 |
| Uruguay | 288 | 1.83 | 9.63 | 0.96 | 28 | 60 | 7.70 | 2.18 | 3.13 | 0.74 | 2.46 | 0.95 | 6.49 | 2.47 |

**Supplementary Table 2**. Correlation matrix of independent and dependent variables

| **Variables** | **(1)** | **(2)** | **(3)** | **(4)** |
| --- | --- | --- | --- | --- |
| (1) Happiness | 1.000 |  |  |  |
| (2) Life Satisfaction | 0.526*^***^* | 1.000 |  |  |
| (3) Age-based status devaluation | -0.121*^***^* | -0.144*^***^* | 1.000 |  |
| (4) Negative age stereotype | -0.055*^***^* | -0.095*^***^* | 0.283*^***^* | 1.000 |

*^*^ p < 0.05, ^**^ p < 0.01, ^***^ p < 0.001*

**Supplementary Figure 1**. Simple Slopes Figures for Significant Interactive Effects

| (a) Significant interactive effects between ageism stereotype and GDP per capita on life satisfaction |
| --- |
| **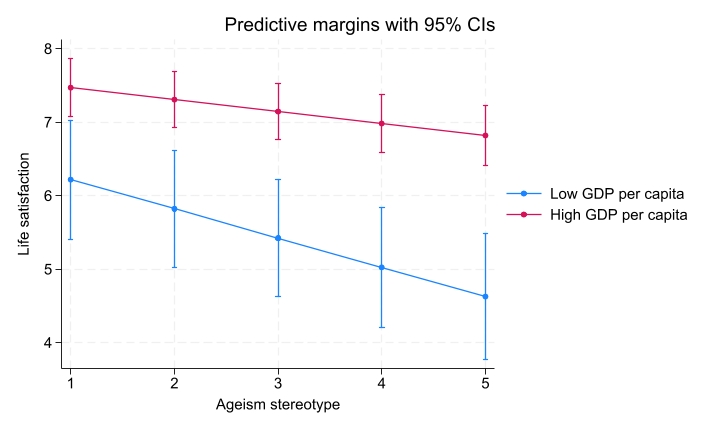** |
| (b) Significant interactive effects between age-based status devaluation and GDP per capita on life satisfaction |
| **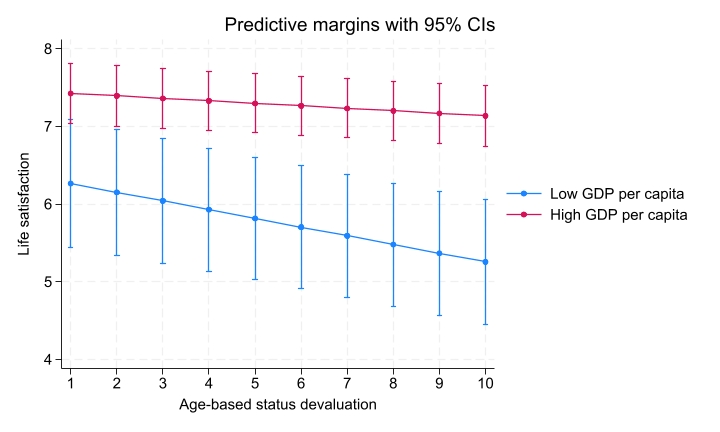** |
| (c) Significant interactive effects between ageism stereotype and stability of governance on life satisfaction |
| **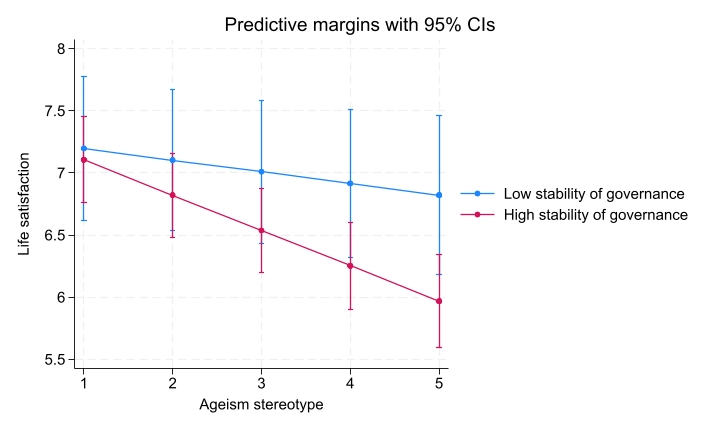** |
| (d) Significant interactive effects between age-based status devaluation and stability of governance on life satisfaction |
| **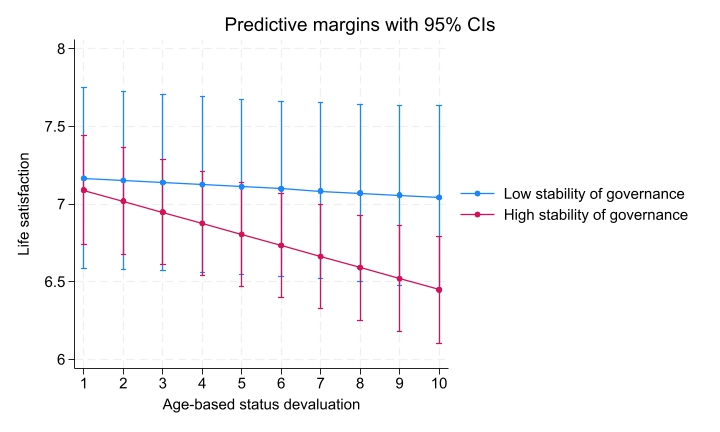** |
| (e) Significant interactive effects between ageism stereotype and long-term orientation on life satisfaction |
| **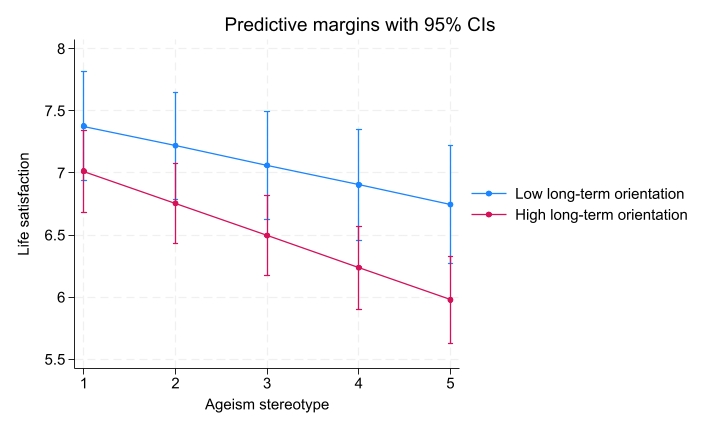** |
| (f) Significant interactive effects between ageism stereotype and stability of governance on happiness |
| **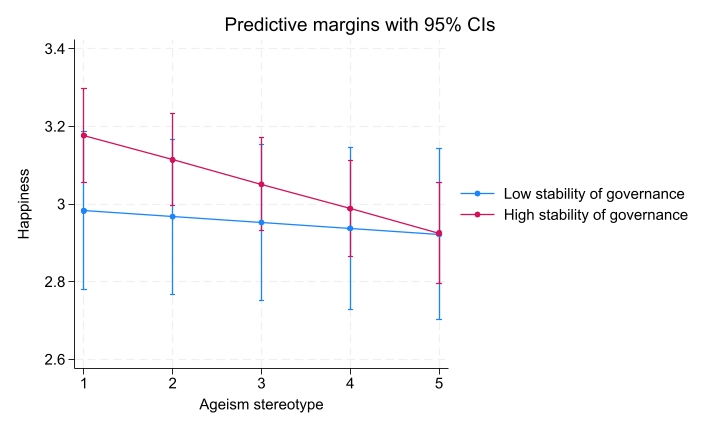** |

**Supplementary Methods. Robustness Check**

*1. Subsample analysis: stricter age-group specification*

To test the robustness of our findings, we conducted a subsample analysis to ensure the results were not unduly influenced by the oldest-old participants. The Socio-Ecological Model (Bronfenbrenner, 1979) suggests that the oldest-old may respond to ageism differently from younger older adults, given their limited access to macro-level resources. We therefore replicated our original analyses on two progressively narrower subsamples: one restricted to respondents aged 55–85, and another to those aged 55–80.

Supplementary Figures 2a and 2b display the coefficients for the main and significant interactive effects on life satisfaction and happiness, respectively, across the full sample and both subsamples. The results demonstrate a high degree of consistency in direction, magnitude, and statistical significance of the coefficients (full models are reported in Supplementary Tables 3-6). This confirms that our findings are robust and not dependent on the inclusion of the oldest-old.

*2. Skewness*

A second robustness check was performed to assess the potential impact of outliers and left-skewness in the well-being distributions. We created two modified versions of the life satisfaction and happiness variables by replacing extremely low values with the values at the 1st and 5th percentiles, respectively. This process mitigates the influence of potential outliers in the lower tail of the distributions.

We then re-estimated our original models using these two modified outcome variables. Supplementary Figure 3a and 3b compare the coefficients for the main and significant interactive effects across the original and adjusted specifications for life satisfaction and happiness, respectively. The results demonstrate a high degree of stability in the direction, magnitude, and statistical significance of the coefficients. The full models, reported in Supplementary Tables 7-10, confirm that our substantive conclusions are robust and not driven by outliers or the skewed nature of the well-being variables.

**Supplementary Figure 2.** Perceived ageism, macro-level sociological factors, and (a) life satisfaction and (b) happiness across three age-trimmed samples

| 1. Life satisfaction | 1. Happiness |
| --- | --- |
|  |  |

**Supplementary Figure 3.** Test for the effect of outliers

| 1. Life satisfaction | 1. Happiness |
| --- | --- |
|  |  |

**Supplementary Table 3.** Perceived ageism, macro-level sociopolitical factors, and life satisfaction (Sample: 55-85 years)

|  | Model 0c | Model 1c | Model 2c | Model 3c |
| --- | --- | --- | --- | --- |
| Negative age stereotype |  | -0.220*** | -1.117*** | -0.220*** |
|  |  | [-0.259,-0.181] | [-1.674,-0.560] | [-0.259,-0.181] |
| Age-based status devaluation |  | -0.053*** | -0.053*** | -0.340** |
|  |  | [-0.067,-0.039] | [-0.067,-0.040] | [-0.544,-0.135] |
| GDP per capita (logged) |  | 0.677** | 0.432# | 0.468# |
|  |  | [0.224,1.130] | [-0.050,0.915] | [-0.007,0.943] |
| Stability of governance |  | -0.172 | 0.052 | -0.005 |
|  |  | [-0.540,0.195] | [-0.347,0.451] | [-0.394,0.385] |
| Long-term orientation |  | -0.010# | -0.005 | -0.008 |
|  |  | [-0.022,0.001] | [-0.018,0.007] | [-0.020,0.004] |
| Individualism |  | 0.000 | 0.000 | 0.003 |
|  |  | [-0.018,0.019] | [-0.020,0.020] | [-0.017,0.023] |
| Negative age stereotype ×GDP per capita (logged) |  |  | 0.105** |  |
|  |  |  | [0.037,0.172] |  |
| Negative age stereotype × Stability of governance |  |  | -0.100** |  |
|  |  |  | [-0.169,-0.032] |  |
| Negative age stereotype × Long-term orientation |  |  | -0.002* |  |
|  |  |  | 0.000 |  |
| Negative age stereotype × Individualism |  |  | [-0.003,0.003] |  |
|  |  |  |  | 0.034** |
| Age-based status devaluation × GDP per capita (logged) |  |  |  | [0.010,0.059] |
|  |  |  |  | -0.032* |
| Age-based status devaluation × Stability of governance |  |  |  | [-0.056,-0.007] |
|  |  |  |  | 0.000 |
| Age-based status devaluation × Long-term orientation |  |  |  | [-0.001,0.000] |
|  |  |  |  | 0.000 |
| Age-based status devaluation × Individualism |  |  |  | [-0.001,0.001] |
|  |  |  | 0.105** |  |
| Age |  | 0.011*** | 0.011*** | 0.011*** |
|  |  | [0.007,0.016] | [0.007,0.016] | [0.007,0.016] |
| Female |  | 0.181*** | 0.181*** | 0.182*** |
|  |  | [0.114,0.248] | [0.113,0.248] | [0.114,0.249] |
| **Marital status (ref: single)** |  |  |  |  |
| Married/cohabited |  | 0.322*** | 0.324*** | 0.326*** |
|  |  | [0.149,0.495] | [0.151,0.497] | [0.153,0.499] |
| divorced/widowed/separated |  | -0.214* | -0.212* | -0.214* |
|  |  | [-0.393,-0.035] | [-0.391,-0.033] | [-0.393,-0.035] |
| **Number of children (ref: no children)** |  |  |  |  |
| has children |  | 0.145* | 0.146* | 0.143* |
|  |  | [0.022,0.269] | [0.023,0.270] | [0.019,0.266] |
| Income level |  | 0.182*** | 0.182*** | 0.182*** |
|  |  | [0.168,0.197] | [0.167,0.197] | [0.167,0.197] |
| **Education (ref: lower than secondary school)** |  |  |  |  |
| Secondary school |  | 0.122** | 0.124** | 0.126** |
|  |  | [0.038,0.207] | [0.039,0.209] | [0.041,0.211] |
| Tertiary education or above |  | 0.218*** | 0.219*** | 0.221*** |
|  |  | [0.116,0.320] | [0.117,0.320] | [0.119,0.322] |
| Unknown |  | 0.068 | 0.075 | 0.078 |
|  |  | [-0.211,0.346] | [-0.204,0.353] | [-0.200,0.357] |
| Constant | 6.727 | -0.320 | 1.752 | 1.405 |
|  | [6.448, 7.006] | [-4.007,3.366] | [-2.175,5.679] | [-2.463,5.273] |
| Variance (Level 2) | 0.854 | 0.548 | 0.555 | 0.544 |
|  | [0.555, 1.313] | [0.354,0.848] | [0.359,0.858] | [0.352,0.842] |
| Variance (Level 1) | 4.512 | 4.159 | 4.154 | 4.156 |
|  | [4.412, 4.614] | [4.067,4.253] | [4.063,4.248] | [4.064,4.250] |
| Log Likelihood | -33666.975 | -33030.322 | -33021.993 | -33024.318 |

95% confidence intervals in brackets

^#^ *p* < 0.1, ^*^ *p* < 0.05, ^**^ *p* < 0.01, ^***^ *p* < 0.001

**Supplementary Table 4.** Perceived ageism, macro-level sociopolitical factors, and life satisfaction (Sample: 55-80 years)

|  | Model 0d | Model 1d | Model 2d | Model 3d |
| --- | --- | --- | --- | --- |
| Negative age stereotype |  | -0.220*** | -1.300*** | -0.219*** |
|  |  | [-0.260,-0.181] | [-1.870,-0.730] | [-0.259,-0.180] |
| Age-based status devaluation |  | -0.053*** | -0.053*** | -0.320** |
|  |  | [-0.067,-0.039] | [-0.067,-0.039] | [-0.529,-0.111] |
| GDP per capita (logged) |  | 0.680** | 0.378 | 0.486* |
|  |  | [0.228,1.133] | [-0.105,0.862] | [0.009,0.962] |
| Stability of governance |  | -0.178 | 0.063 | 0.000 |
|  |  | [-0.546,0.189] | [-0.337,0.463] | [-0.390,0.390] |
| Long-term orientation |  | -0.010# | -0.005 | -0.008 |
|  |  | [-0.022,0.002] | [-0.018,0.007] | [-0.020,0.004] |
| Individualism |  | 0.000 | 0.002 | 0.003 |
|  |  | [-0.019,0.019] | [-0.018,0.022] | [-0.017,0.023] |
| Negative age stereotype ×GDP per capita (logged) |  |  | 0.129*** |  |
|  |  |  | [0.060,0.198] |  |
| Negative age stereotype × Stability of governance |  |  | -0.108** |  |
|  |  |  | [-0.178,-0.038] |  |
| Negative age stereotype × Long-term orientation |  |  | -0.002* |  |
|  |  |  | [-0.004,-0.000] |  |
| Negative age stereotype × Individualism |  |  | -0.001 |  |
|  |  |  | [-0.004,0.002] |  |
| Age-based status devaluation × GDP per capita (logged) |  |  |  | 0.032* |
|  |  |  |  | [0.007,0.057] |
| Age-based status devaluation × Stability of governance |  |  |  | -0.034** |
|  |  |  |  | [-0.059,-0.009] |
| Age-based status devaluation × Long-term orientation |  |  |  | 0.000 |
|  |  |  |  | [-0.001,0.000] |
| Age-based status devaluation × Individualism |  |  |  | 0.000 |
|  |  |  |  | [-0.001,0.001] |
| Age |  | 0.013*** | 0.013*** | 0.013*** |
|  |  | [0.008,0.018] | [0.008,0.018] | [0.008,0.018] |
| Female |  | 0.186*** | 0.186*** | 0.187*** |
|  |  | [0.118,0.255] | [0.117,0.255] | [0.119,0.256] |
| **Marital status (ref: single)** |  |  |  |  |
| Married/cohabited |  | 0.304*** | 0.306*** | 0.308*** |
|  |  | [0.128,0.480] | [0.130,0.482] | [0.132,0.484] |
| divorced/widowed/separated |  | -0.235* | -0.232* | -0.234* |
|  |  | [-0.417,-0.053] | [-0.414,-0.050] | [-0.416,-0.052] |
| **Number of children (ref: no children)** |  |  |  |  |
| has children |  | 0.148* | 0.150* | 0.145* |
|  |  | [0.021,0.275] | [0.023,0.277] | [0.018,0.272] |
| Income level |  | 0.181*** | 0.181*** | 0.181*** |
|  |  | [0.167,0.196] | [0.166,0.196] | [0.166,0.196] |
| **Education (ref: lower than secondary school)** |  |  |  |  |
| Secondary school |  | 0.132** | 0.134** | 0.136** |
|  |  | [0.045,0.219] | [0.047,0.220] | [0.049,0.222] |
| Tertiary education or above |  | 0.232*** | 0.233*** | 0.236*** |
|  |  | [0.128,0.336] | [0.129,0.338] | [0.132,0.340] |
| Unknown |  | 0.075 | 0.085 | 0.084 |
|  |  | [-0.214,0.363] | [-0.204,0.373] | [-0.204,0.373] |
| Constant | 6.728 | -0.443 | 2.059 | 1.166 |
|  | [6.450, 7.005] | [-4.129,3.243] | [-1.881,5.999] | [-2.714,5.046] |
| Variance (Level 2) | 0.845 | 0.547 | 0.554 | 0.544 |
|  | [0.549, 1.299] | [0.353,0.846] | [0.358,0.858] | [0.351,0.842] |
| Variance (Level 1) | 4.473 | 4.119 | 4.114 | 4.116 |
|  | [4.371, 4.576] | [4.026,4.215] | [4.021,4.209] | [4.023,4.211] |
| Log Likelihood | -32029.037 | -31415.749 | -31405.718 | -31409.565 |

95% confidence intervals in brackets

^#^ *p* < 0.1, ^*^ *p* < 0.05, ^**^ *p* < 0.01, ^***^ *p* < 0.001

**Supplementary Table 5.** Perceived ageism, macro-level sociopolitical factors, and happiness (Sample: 55-85 years)

|  | Model 0e | Model 1e | Model 2e | Model 3e |
| --- | --- | --- | --- | --- |
| Negative age stereotype |  | -0.046*** | -0.152 | -0.045*** |
|  |  | [-0.059,-0.033] | [-0.336,0.032] | [-0.058,-0.032] |
| Age-based status devaluation |  | -0.012*** | -0.012*** | -0.002 |
|  |  | [-0.017,-0.007] | [-0.017,-0.007] | [-0.070,0.065] |
| GDP per capita (logged) |  | 0.166* | 0.134 | 0.171* |
|  |  | [0.005,0.326] | [-0.035,0.303] | [0.003,0.340] |
| Stability of governance |  | 0.069 | 0.120# | 0.108 |
|  |  | [-0.061,0.198] | [-0.020,0.259] | [-0.030,0.246] |
| Long-term orientation |  | -0.001 | -0.001 | -0.001 |
|  |  | [-0.005,0.003] | [-0.005,0.004] | [-0.006,0.003] |
| Individualism |  | -0.006# | -0.005 | -0.006 |
|  |  | [-0.012,0.001] | [-0.012,0.002] | [-0.013,0.001] |
| Negative age stereotype ×GDP per capita (logged) |  |  | 0.014 |  |
|  |  |  | [-0.009,0.036] |  |
| Negative age stereotype × Stability of governance |  |  | -0.023* |  |
|  |  |  | [-0.046,-0.000] |  |
| Negative age stereotype × Long-term orientation |  |  | 0.000 |  |
|  |  |  | [-0.001,0.001] |  |
| Negative age stereotype × Individualism |  |  | 0.000 |  |
|  |  |  | [-0.001,0.001] |  |
| Age-based status devaluation × GDP per capita (logged) |  |  |  | -0.001 |
|  |  |  |  | [-0.009,0.007] |
| Age-based status devaluation × Stability of governance |  |  |  | -0.008# |
|  |  |  |  | [-0.016,0.000] |
| Age-based status devaluation × Long-term orientation |  |  |  | 0.000 |
|  |  |  |  | [-0.000,0.000] |
| Age-based status devaluation × Individualism |  |  |  | 0.000 |
|  |  |  |  | [-0.000,0.000] |
| Age |  | 0.003*** | 0.003*** | 0.003*** |
|  |  | [0.001,0.004] | [0.001,0.004] | [0.001,0.004] |
| Female |  | 0.065*** | 0.065*** | 0.065*** |
|  |  | [0.042,0.087] | [0.043,0.087] | [0.043,0.087] |
| Marital status (ref: single) |  |  |  |  |
| Married/cohabited |  | 0.144*** | 0.144*** | 0.145*** |
|  |  | [0.086,0.201] | [0.087,0.201] | [0.088,0.202] |
| divorced/widowed/separated |  | -0.080** | -0.080** | -0.079** |
|  |  | [-0.139,-0.021] | [-0.138,-0.021] | [-0.138,-0.020] |
| Number of children (ref: no children) |  |  |  |  |
| has children |  | 0.121*** | 0.121*** | 0.119*** |
|  |  | [0.080,0.162] | [0.080,0.162] | [0.078,0.160] |
| Income level |  | 0.040*** | 0.040*** | 0.040*** |
|  |  | [0.035,0.044] | [0.035,0.044] | [0.035,0.044] |
| Education (ref: lower than secondary school) |  |  |  |  |
| Secondary school |  | 0.031* | 0.032* | 0.033* |
|  |  | [0.003,0.059] | [0.004,0.060] | [0.005,0.061] |
| Tertiary education or above |  | 0.080*** | 0.082*** | 0.083*** |
|  |  | [0.047,0.114] | [0.049,0.116] | [0.050,0.117] |
| Unknown |  | 0.012 | 0.015 | 0.009 |
|  |  | [-0.080,0.103] | [-0.077,0.107] | [-0.083,0.101] |
| Constant | 3.031 | 1.381* | 1.627* | 1.335# |
|  | [2.939, 3.124] | [0.079,2.683] | [0.253,3.001] | [-0.038,2.709] |
| Variance (Level 2) | 0.094 | 0.069 | 0.069 | 0.07 |
|  | [0.061, 0.145] | [0.045,0.106] | [0.045,0.107] | [0.045,0.108] |
| Variance (Level 1) | 0.479 | 0.452 | 0.452 | 0.452 |
|  | [0.468, 0.490] | [0.442,0.462] | [0.442,0.462] | [0.442,0.462] |
| Log Likelihood | -16333.711 | -15880.837 | -15876.359 | -15874.028 |

95% confidence intervals in brackets

^#^ *p* < 0.1, ^*^ *p* < 0.05, ^**^ *p* < 0.01, ^***^ *p* < 0.001

**Supplementary Table 6.** Perceived ageism, macro-level sociopolitical factors, and happiness (Sample: 55-80 years)

|  | Model 0f | Model 1f | Model 2f | Model 3f |
| --- | --- | --- | --- | --- |
| Negative age stereotype |  | -0.046*** | -0.176# | -0.046*** |
|  |  | [-0.060,-0.033] | [-0.364,0.013] | [-0.059,-0.033] |
| Age-based status devaluation |  | -0.011*** | -0.011*** | -0.004 |
|  |  | [-0.016,-0.006] | [-0.016,-0.006] | [-0.073,0.065] |
| GDP per capita (logged) |  | 0.167* | 0.127 | 0.170* |
|  |  | [0.006,0.328] | [-0.043,0.297] | [0.000,0.340] |
| Stability of governance |  | 0.064 | 0.115 | 0.108 |
|  |  | [-0.066,0.194] | [-0.026,0.255] | [-0.031,0.247] |
| Long-term orientation |  | -0.001 | -0.001 | -0.001 |
|  |  | [-0.005,0.003] | [-0.005,0.004] | [-0.006,0.003] |
| Individualism |  | -0.006# | -0.005 | -0.005 |
|  |  | [-0.012,0.001] | [-0.012,0.003] | [-0.012,0.002] |
| Negative age stereotype ×GDP per capita (logged) |  |  | 0.017 |  |
|  |  |  | [-0.006,0.040] |  |
| Negative age stereotype × Stability of governance |  |  | -0.023# |  |
|  |  |  | [-0.046,0.000] |  |
| Negative age stereotype × Long-term orientation |  |  | 0.000 |  |
|  |  |  | [-0.001,0.001] |  |
| Negative age stereotype × Individualism |  |  | 0.000 |  |
|  |  |  | [-0.002,0.001] |  |
| Age-based status devaluation × GDP per capita (logged) |  |  |  | -0.001 |
|  |  |  |  | [-0.009,0.007] |
| Age-based status devaluation × Stability of governance |  |  |  | -0.009* |
|  |  |  |  | [-0.017,-0.001] |
| Age-based status devaluation × Long-term orientation |  |  |  | 0.000 |
|  |  |  |  | [-0.000,0.000] |
| Age-based status devaluation × Individualism |  |  |  | 0.000 |
|  |  |  |  | [-0.000,0.000] |
| Age |  | 0.003** | 0.003** | 0.003** |
|  |  | [0.001,0.004] | [0.001,0.004] | [0.001,0.004] |
| Female |  | 0.072*** | 0.072*** | 0.072*** |
|  |  | [0.049,0.094] | [0.049,0.095] | [0.050,0.095] |
| Marital status (ref: single) |  |  |  |  |
| Married/cohabited |  | 0.155*** | 0.156*** | 0.156*** |
|  |  | [0.097,0.213] | [0.098,0.214] | [0.098,0.215] |
| divorced/widowed/separated |  | -0.066* | -0.065* | -0.064* |
|  |  | [-0.126,-0.006] | [-0.125,-0.005] | [-0.124,-0.004] |
| Number of children (ref: no children) |  |  |  |  |
| has children |  | 0.115*** | 0.115*** | 0.112*** |
|  |  | [0.073,0.157] | [0.073,0.157] | [0.070,0.154] |
| Income level |  | 0.039*** | 0.039*** | 0.039*** |
|  |  | [0.034,0.044] | [0.034,0.044] | [0.034,0.044] |
| Education (ref: lower than secondary school) |  |  |  |  |
| Secondary school |  | 0.035* | 0.035* | 0.036* |
|  |  | [0.006,0.063] | [0.007,0.064] | [0.008,0.065] |
| Tertiary education or above |  | 0.085*** | 0.087*** | 0.089*** |
|  |  | [0.051,0.120] | [0.053,0.122] | [0.054,0.123] |
| Unknown |  | 0.013 | 0.016 | 0.010 |
|  |  | [-0.083,0.108] | [-0.079,0.112] | [-0.085,0.106] |
| Constant | 3.031 | 1.482 | 1.664 | 1.431 |
|  | [2.939, 3.123] | [0.158,2.806] | [0.278,3.049] | [0.044,2.819] |
| Variance (Level 2) | 0.094 | 0.069 | 0.07 | 0.071 |
|  | [0.061, 0.144] | [0.045,0.107] | [0.045,0.108] | [0.046,0.109] |
| Variance (Level 1) | 0.476 | 0.449 | 0.449 | 0.449 |
|  | [0.465, 0.487] | [0.439,0.460] | [0.439,0.459] | [0.439,0.459] |
| Log Likelihood | -15524.353 | -15095.226 | -15090.371 | -15087.012 |

95% confidence intervals in brackets

^#^ *p* < 0.1, ^*^ *p* < 0.05, ^**^ *p* < 0.01, ^***^ *p* < 0.001

**Supplementary Table 7.** Perceived ageism, macro-level sociopolitical factors, and life satisfaction (Pctile.1)

|  | Model 0g | Model 1g | Model 2g | Model 3g |
| --- | --- | --- | --- | --- |
| Negative age stereotype |  | -0.226^***^ | -1.087^***^ | -0.226^***^ |
|  |  | [-0.265,-0.188] | [-1.638,-0.536] | [-0.264,-0.188] |
| Age-based status devaluation |  | -0.052*** | -0.053*** | -0.338** |
|  |  | [-0.066,-0.039] | [-0.066,-0.039] | [-0.540,-0.135] |
| GDP per capita (logged) |  | 0.686^**^ | 0.449^#^ | 0.475^#^ |
|  |  | [0.227,1.145] | [-0.038,0.936] | [-0.005,0.955] |
| Stability of governance |  | -0.165 | 0.047 | -0.012 |
|  |  | [-0.537,0.207] | [-0.355,0.450] | [-0.405,0.381] |
| Long-term orientation |  | -0.010^#^ | -0.006 | -0.007 |
|  |  | [-0.022,0.001] | [-0.018,0.007] | [-0.020,0.005] |
| Individualism |  | 0.000 | 0.000 | 0.003 |
|  |  | [-0.019,0.019] | [-0.020,0.020] | [-0.017,0.023] |
| Negative age stereotype × GDP per capita (logged) |  |  | 0.101** |  |
|  |  |  | [0.035,0.168] |  |
| Negative age stereotype × Stability of governance |  |  | -0.095^**^ |  |
|  |  |  | [-0.163,-0.027] |  |
| Negative age stereotype × Long-term orientation |  |  | -0.002* |  |
|  |  |  | [-0.004,-0.000] |  |
| Negative age stereotype × Individualism |  |  | 0.000 |  |
|  |  |  | [-0.003,0.003] |  |
| Age-based status devaluation × GDP per capita (logged) |  |  |  | 0.035^**^ |
|  |  |  |  | [0.010,0.059] |
| Age-based status devaluation × Stability of governance |  |  |  | -0.029^*^ |
|  |  |  |  | [-0.053,-0.004] |
| Age-based status devaluation × Long-term orientation |  |  |  | 0.000 |
|  |  |  |  | [-0.001,0.000] |
| Age-based status devaluation × Individualism |  |  |  | 0.000 |
|  |  |  |  | [-0.001,0.001] |
| Age |  | 0.009^***^ | 0.009^***^ | 0.009^***^ |
|  |  | [0.005,0.014] | [0.005,0.014] | [0.005,0.014] |
| Female |  | 0.175^***^ | 0.175^***^ | 0.176^***^ |
|  |  | [0.108,0.241] | [0.108,0.241] | [0.109,0.242] |
| Marital status (ref: single) |  |  |  |  |
| Married/cohabited |  | 0.336^***^ | 0.338^***^ | 0.340^***^ |
|  |  | [0.165,0.507] | [0.167,0.509] | [0.169,0.511] |
| divorced/widowed/separated |  | -0.195^*^ | -0.193^*^ | -0.194^*^ |
|  |  | [-0.371,-0.018] | [-0.369,-0.016] | [-0.371,-0.018] |
| Number of children (ref: no children) |  |  |  |  |
| has children |  | 0.147^*^ | 0.149^*^ | 0.145^*^ |
|  |  | [0.026,0.269] | [0.027,0.270] | [0.023,0.267] |
| Income level |  | 0.178^***^ | 0.178^***^ | 0.178^***^ |
|  |  | [0.164,0.193] | [0.164,0.193] | [0.164,0.193] |
| Education (ref: lower than secondary school) |  |  |  |  |
| Secondary school |  | 0.126^**^ | 0.128^**^ | 0.129^**^ |
|  |  | [0.042,0.210] | [0.044,0.211] | [0.046,0.213] |
| Tertiary education or above |  | 0.223^***^ | 0.224^***^ | 0.225^***^ |
|  |  | [0.123,0.324] | [0.124,0.325] | [0.124,0.326] |
| Unknown |  | 0.113 | 0.120 | 0.124 |
|  |  | [-0.160,0.387] | [-0.153,0.393] | [-0.149,0.397] |
| Constant | 6.727 | -0.240 | 1.749 | 1.479 |
|  | [6.446, 7.009] | [-3.970,3.490] | [-2.213,5.711] | [-2.424,5.382] |
| Variance (Level 2) | 0.869 | 0.563 | 0.569 | 0.558 |
|  | [0.565, 1.336] | [0.364,0.870] | [0.368,0.880] | [0.361,0.863] |
| Variance (Level 1) | 4.468 | 4.122 | 4.118 | 4.119 |
|  | [4.370, 4.568] | [4.032,4.215] | [4.028,4.210] | [4.029,4.212] |
| Log Likelihood | -34110.356 | -33471.300 | -33463.300 | -33465.300 |

95% confidence intervals in brackets

^#^ *p* < 0.1, ^*^ *p* < 0.05, ^**^ *p* < 0.01, ^***^ *p* < 0.001

**Supplementary Table 8.** Perceived ageism, macro-level sociopolitical factors, and life satisfaction (Pctile.5)

|  | Model 0h | Model 1h | Model 2h | Model 3h |
| --- | --- | --- | --- | --- |
| Negative age stereotype |  | -0.223^***^ | -1.040^***^ | -0.222^***^ |
|  |  | [-0.259,-0.186] | [-1.563,-0.516] | [-0.259,-0.186] |
| Age-based status devaluation |  | -0.050^***^ | -0.050^***^ | -0.327^***^ |
|  |  | [-0.063,-0.037] | [-0.063,-0.037] | [-0.519,-0.135] |
| GDP per capita (logged) |  | 0.692^**^ | 0.467^#^ | 0.483^#^ |
|  |  | [0.222,1.163] | [-0.029,0.963] | [-0.006,0.972] |
| Stability of governance |  | -0.170 | 0.028 | -0.030 |
|  |  | [-0.551,0.211] | [-0.380,0.437] | [-0.429,0.370] |
| Long-term orientation |  | -0.011^#^ | -0.006 | -0.007 |
|  |  | [-0.023,0.002] | [-0.019,0.007] | [-0.020,0.005] |
| Individualism |  | 0.000 | 0.000 | 0.003 |
|  |  | [-0.020,0.019] | [-0.021,0.020] | [-0.018,0.023] |
| Negative age stereotype × GDP per capita (logged) |  |  | 0.096^**^ |  |
|  |  |  | [0.033,0.160] |  |
| Negative age stereotype × Stability of governance |  |  | -0.089^**^ |  |
|  |  |  | [-0.153,-0.024] |  |
| Negative age stereotype × Long-term orientation |  |  | -0.002^#^ |  |
|  |  |  | [-0.004,0.000] |  |
| Negative age stereotype × Individualism |  |  | 0.000 |  |
|  |  |  | [-0.003,0.003] |  |
| Age-based status devaluation × GDP per capita (logged) |  |  |  | 0.034^**^ |
|  |  |  |  | [0.011,0.058] |
| Age-based status devaluation × Stability of governance |  |  |  | -0.026^*^ |
|  |  |  |  | [-0.050,-0.003] |
| Age-based status devaluation × Long-term orientation |  |  |  | -0.001 |
|  |  |  |  | [-0.001,0.000] |
| Age-based status devaluation × Individualism |  |  |  | 0.000 |
|  |  |  |  | [-0.001,0.001] |
| Age |  | 0.009^***^ | 0.009^***^ | 0.009^***^ |
|  |  | [0.005,0.013] | [0.005,0.014] | [0.005,0.013] |
| Female |  | 0.170^***^ | 0.170^***^ | 0.171^***^ |
|  |  | [0.107,0.234] | [0.107,0.233] | [0.108,0.234] |
| Marital status (ref: single) |  |  |  |  |
| Married/cohabited |  | 0.308^***^ | 0.310^***^ | 0.312^***^ |
|  |  | [0.146,0.470] | [0.148,0.472] | [0.150,0.474] |
| divorced/widowed/separated |  | -0.190^*^ | -0.188^*^ | -0.190^*^ |
|  |  | [-0.358,-0.023] | [-0.356,-0.021] | [-0.357,-0.022] |
| Number of children (ref: no children) |  |  |  |  |
| has children |  | 0.139^*^ | 0.140^*^ | 0.137^*^ |
|  |  | [0.023,0.254] | [0.025,0.256] | [0.021,0.252] |
| Income level |  | 0.168^***^ | 0.168^***^ | 0.168^***^ |
|  |  | [0.155,0.182] | [0.155,0.182] | [0.155,0.182] |
| Education (ref: lower than secondary school) |  |  |  |  |
| Secondary school |  | 0.121^**^ | 0.123^**^ | 0.125^**^ |
|  |  | [0.042,0.201] | [0.043,0.202] | [0.045,0.204] |
| Tertiary education or above |  | 0.218^***^ | 0.219^***^ | 0.220^***^ |
|  |  | [0.122,0.313] | [0.123,0.314] | [0.124,0.315] |
| Unknown |  | 0.114 | 0.121 | 0.125 |
|  |  | [-0.145,0.373] | [-0.138,0.380] | [-0.135,0.384] |
| Constant | 6.776 | -0.181 | 1.708 | 1.493 |
|  | [6.491, 7.061] | [-4.003,3.642] | [-2.321,5.737] | [-2.480,5.466] |
| Variance (Level 2) | 0.895 | 0.594 | 0.600 | 0.589 |
|  | [0.582, 1.374] | [0.385,0.917] | [0.389,0.926] | [0.382,0.909] |
| Variance (Level 1) | 4.026 | 3.714 | 3.710 | 3.711 |
|  | [3.938, 4.116] | [3.633,3.797] | [3.629,3.793] | [3.630,3.794] |
| Log Likelihood | -33296.339 | -32655.900 | -32648.200 | -32649.500 |

95% confidence intervals in brackets

^#^ *p* < 0.1, ^*^ *p* < 0.05, ^**^ *p* < 0.01, ^***^ *p* < 0.001

**Supplementary Table 9.** Perceived ageism, macro-level sociopolitical factors, and happiness (Pctile.1)

|  | Model 0j | Model 1j | Model 2j | Model 3j |
| --- | --- | --- | --- | --- |
| Negative age stereotype |  | -0.047^***^ | -0.166^#^ | -0.046^***^ |
|  |  | [-0.059,-0.034] | [-0.347,0.015] | [-0.059,-0.034] |
| Age-based status devaluation |  | -0.012^***^ | -0.012^***^ | -0.007 |
|  |  | [-0.016,-0.008] | [-0.016,-0.007] | [-0.073,0.060] |
| GDP per capita (logged) |  | 0.167^*^ | 0.131 | 0.169^#^ |
|  |  | [0.006,0.329] | [-0.039,0.301] | [-0.001,0.339] |
| Stability of governance |  | 0.067 | 0.117 | 0.104 |
|  |  | [-0.064,0.198] | [-0.023,0.257] | [-0.035,0.243] |
| Long-term orientation |  | -0.001 | -0.001 | -0.001 |
|  |  | [-0.005,0.003] | [-0.005,0.004] | [-0.006,0.003] |
| Individualism |  | -0.006^#^ | -0.005 | -0.006 |
|  |  | [-0.013,0.001] | [-0.012,0.002] | [-0.013,0.001] |
| Negative age stereotype ×GDP per capita (logged) |  |  | 0.016 |  |
|  |  |  | [-0.006,0.038] |  |
| Negative age stereotype × Stability of governance |  |  | -0.022^#^ |  |
|  |  |  | [-0.045,0.000] |  |
| Negative age stereotype × Long-term orientation |  |  | 0.000 |  |
|  |  |  | [-0.001,0.000] |  |
| Negative age stereotype × Individualism |  |  | 0.000 |  |
|  |  |  | [-0.001,0.001] |  |
| Age-based status devaluation × GDP per capita (logged) |  |  |  | 0.000 |
|  |  |  |  | [-0.008,0.008] |
| Age-based status devaluation × Stability of governance |  |  |  | -0.008^#^ |
|  |  |  |  | [-0.016,0.000] |
| Age-based status devaluation × Long-term orientation |  |  |  | 0.000 |
|  |  |  |  | [-0.000,0.000] |
| Age-based status devaluation × Individualism |  |  |  | 0.000 |
|  |  |  |  | [-0.000,0.000] |
| Age |  | 0.003^***^ | 0.003^***^ | 0.003^***^ |
|  |  | [0.001,0.004] | [0.001,0.004] | [0.001,0.004] |
| Female |  | 0.063^***^ | 0.063^***^ | 0.063^***^ |
|  |  | [0.041,0.085] | [0.041,0.085] | [0.041,0.085] |
| Marital status (ref: single) |  |  |  |  |
| Married/cohabited |  | 0.145^***^ | 0.145^***^ | 0.146^***^ |
|  |  | [0.088,0.201] | [0.089,0.201] | [0.090,0.202] |
| divorced/widowed/separated |  | -0.075^*^ | -0.074^*^ | -0.073^*^ |
|  |  | [-0.133,-0.017] | [-0.132,-0.016] | [-0.131,-0.015] |
| Number of children (ref: no children) |  |  |  |  |
| has children |  | 0.120^***^ | 0.120^***^ | 0.118^***^ |
|  |  | [0.080,0.160] | [0.080,0.160] | [0.078,0.158] |
| Income level |  | 0.039^***^ | 0.038^***^ | 0.039^***^ |
|  |  | [0.034,0.043] | [0.034,0.043] | [0.034,0.043] |
| Education (ref: lower than secondary school) |  |  |  |  |
| Secondary school |  | 0.032^*^ | 0.033^*^ | 0.033^*^ |
|  |  | [0.004,0.059] | [0.005,0.060] | [0.006,0.061] |
| Tertiary education or above |  | 0.081^***^ | 0.083^***^ | 0.084^***^ |
|  |  | [0.048,0.114] | [0.050,0.116] | [0.051,0.117] |
| Unknown |  | 0.020 | 0.023 | 0.018 |
|  |  | [-0.070,0.110] | [-0.067,0.113] | [-0.072,0.108] |
| Constant | 3.034 | 1.372 | 1.648 | 1.351 |
|  | [2.941, 3.127] | [0.059,2.684] | [0.266,3.030] | [-0.030,2.733] |
| Variance (Level 2) | 0.095 | 0.070 | 0.070 | 0.071 |
|  | [0.062, 0.147] | [0.045,0.108] | [0.046,0.109] | [0.046,0.110] |
| Variance (Level 1) | 0.471 | 0.445 | 0.445 | 0.445 |
|  | [0.461, 0.482] | [0.436,0.455] | [0.435,0.455] | [0.435,0.455] |
| Log Likelihood | -16460.569 | -16007.300 | -16002.500 | -16001.000 |

95% confidence intervals in brackets

^#^ *p* < 0.1, ^*^ *p* < 0.05, ^**^ *p* < 0.01, ^***^ *p* < 0.001

**Supplementary Table 10.** Perceived ageism, macro-level sociopolitical factors, and happiness (Pctile.5)

|  | Model 0k | Model 1k | Model 2k | Model 3k |
| --- | --- | --- | --- | --- |
| Negative age stereotype |  | -0.045^***^ | -0.124 | -0.045^***^ |
|  |  | [-0.057,-0.033] | [-0.295,0.047] | [-0.056,-0.033] |
| Age-based status devaluation |  | -0.011^***^ | -0.011^***^ | -0.001 |
|  |  | [-0.016,-0.007] | [-0.016,-0.007] | [-0.063,0.062] |
| GDP per capita (logged) |  | 0.163^#^ | 0.137 | 0.167^#^ |
|  |  | [-0.000,0.326] | [-0.034,0.307] | [-0.004,0.337] |
| Stability of governance |  | 0.068 | 0.108 | 0.099 |
|  |  | [-0.064,0.200] | [-0.032,0.248] | [-0.041,0.238] |
| Long-term orientation |  | -0.001 | -0.001 | -0.001 |
|  |  | [-0.005,0.003] | [-0.005,0.004] | [-0.006,0.003] |
| Individualism |  | -0.006^#^ | -0.005 | -0.006 |
|  |  | [-0.013,0.001] | [-0.012,0.002] | [-0.013,0.002] |
| Negative age stereotype ×GDP per capita (logged) |  |  | 0.011 |  |
|  |  |  | [-0.009,0.032] |  |
| Negative age stereotype × Stability of governance |  |  | -0.018^#^ |  |
|  |  |  | [-0.039,0.003] |  |
| Negative age stereotype × Long-term orientation |  |  | 0.000 |  |
|  |  |  | [-0.001,0.000] |  |
| Negative age stereotype × Individualism |  |  | 0.000 |  |
|  |  |  | [-0.001,0.001] |  |
| Age-based status devaluation × GDP per capita (logged) |  |  |  | -0.001 |
|  |  |  |  | [-0.008,0.007] |
| Age-based status devaluation × Stability of governance |  |  |  | -0.006^#^ |
|  |  |  |  | [-0.014,0.001] |
| Age-based status devaluation × Long-term orientation |  |  |  | 0.000 |
|  |  |  |  | [-0.000,0.000] |
| Age-based status devaluation × Individualism |  |  |  | 0.000 |
|  |  |  |  | [-0.000,0.000] |
| Age |  | 0.003^***^ | 0.003^***^ | 0.003^***^ |
|  |  | [0.001,0.004] | [0.001,0.004] | [0.001,0.004] |
| Female |  | 0.059^***^ | 0.059^***^ | 0.059^***^ |
|  |  | [0.038,0.079] | [0.038,0.080] | [0.038,0.080] |
| Marital status (ref: single) |  |  |  |  |
| Married/cohabited |  | 0.134^***^ | 0.134^***^ | 0.135^***^ |
|  |  | [0.081,0.187] | [0.081,0.187] | [0.082,0.188] |
| divorced/widowed/separated |  | -0.069^*^ | -0.068^*^ | -0.068^*^ |
|  |  | [-0.124,-0.014] | [-0.123,-0.014] | [-0.122,-0.013] |
| Number of children (ref: no children) |  |  |  |  |
| has children |  | 0.112^***^ | 0.113^***^ | 0.111^***^ |
|  |  | [0.075,0.150] | [0.075,0.151] | [0.073,0.148] |
| Income level |  | 0.035^***^ | 0.035^***^ | 0.035^***^ |
|  |  | [0.031,0.040] | [0.031,0.040] | [0.031,0.040] |
| Education (ref: lower than secondary school) |  |  |  |  |
| Secondary school |  | 0.027^*^ | 0.028^*^ | 0.028^*^ |
|  |  | [0.001,0.053] | [0.002,0.054] | [0.002,0.054] |
| Tertiary education or above |  | 0.073^***^ | 0.075^***^ | 0.076^***^ |
|  |  | [0.042,0.104] | [0.044,0.106] | [0.045,0.107] |
| Unknown |  | 0.018 | 0.021 | 0.016 |
|  |  | [-0.067,0.103] | [-0.064,0.106] | [-0.069,0.101] |
| Constant | 3.055 | 1.482 | 1.664 | 1.431 |
|  | [2.962, 3.148] | [0.158,2.806] | [0.278,3.049] | [0.044,2.819] |
| Variance (Level 2) | 0.095 | 0.072 | 0.072 | 0.073 |
|  | [0.062, 0.146] | [0.046,0.110] | [0.047,0.111] | [0.047,0.112] |
| Variance (Level 1) | 0.420 | 0.397 | 0.397 | 0.397 |
|  | [0.410, 0.429] | [0.389,0.406] | [0.388,0.406] | [0.388,0.406] |
| Log Likelihood | -15550.065 | -15115.200 | -15110.500 | -15108.300 |

95% confidence intervals in brackets

^#^ *p* < 0.1, ^*^ *p* < 0.05, ^**^ *p* < 0.01, ^***^ *p* < 0.001
